# Obesity and cardiovascular disease: An executive document on pathophysiological and clinical links promoted by the Italian Society of Cardiovascular Prevention (SIPREC)

**DOI:** 10.3389/fcvm.2023.1136340

**Published:** 2023-03-13

**Authors:** Massimo Volpe, Giovanna Gallo

**Affiliations:** ^1^ Department of Clinical and Molecular Medicine, Sapienza University of Rome, Rome, Italy; ^2^ IRCCS San Raffaele, Rome, Italy

**Keywords:** obesity, cardiovascular prevention, cardiovascular disease, chronic non-communicable disease, pharmacological treatment

## Abstract

The prevalence of obesity worldwide has increased in recent decades not only among adults, but also in children and adolescents. This phenomenon contributes to an increased risk of cardiovascular diseases (CVD), also after the adjustment for conventional risk factors such as hypertension, diabetes and dyslipidemia. Indeed, obesity contributes to the development of insulin resistance, endothelial dysfunction, sympathetic nervous system activation, increased vascular resistance and inflammatory and prothrombotic state which promote the incidence of major cardiovascular events. On the basis of this evidence, in 2021 obesity has been acknowledged as a definite pathological identity and identified as a recurrent, chronic non-communicable disease. Therapeutic strategies for the pharmacological treatment of obesity include the combination of naltrexone and bupropione and the lipase inhibitor orlistat and they have been recently implemented with the glucagon like peptide-1 receptor agonists semaglutide and liraglutide, which have produced positive and sustained effects on body weight reduction. If drug interventions are not effective, bariatric surgery may be considered, representing an efficacious treatment option for extreme obesity or obesity with comorbidities. The present executive paper is aimed to increase knowledge on the relationships between obesity and CVD, to raise the perception of this condition which is currently insufficient and to support the clinical practice management.

## Introduction

In the last decades the prevalence of obesity [defined as body mass index (BMI) ≥ 30 Kg/m^2^] has enormously increased, becoming a real epidemic which involves hundred millions of people worldwide. The Global Burden of Disease Obesity Collaborations estimated that in 2017 about 603.7 million of adults were affected by obesity, doubling as compared to the early ‘80s (1). These numbers, although often underestimated, become even more impressive when overweight subjects are considered, reaching 3 billions people worldwide (2). Moreover, the World Health Organization has estimated that 18% of males and 21% of females will be obese by 2025, with 40% of these individuals reaching BMI levels ≥25 Kg/m^2^. This phenomenon is worringly widespreading also among children and adolescents irrespective of age, accounting for 40 and 340 million of obese and overweight subjects, respectively, with a 47% increase in the last thirty years (3).

Although a large body of evidence lends support to the pathophysiological link between obesity and cardiovascular (CV) diseases (CVD), obesity has been considered for a long time a minor risk factor or even a simple amplifier of the recognized role of the other well established CV risk factors, such as hypertension, diabetes and dyslipidemias. Only in 2021 obesity has been finally acknowledged as a definite pathological identity and identified as a recurrent, chronic non-communicable disease (4).

The Italian Society of Cardiovascular Prevention (SIPREC) with the active contributions of experts from different Italian Scientific Societies (see Appendix) has recently produced an updated multisciplinary consensus document to analyze and discuss evidence about the role of obesity in the development of CV events as well as the available pharmacological and non-pharmacological strategies to manage this condition. The purpose of this consensus document was to provide an integrated tool to afford all the main aspects of the cause-effect relationship between obesity and CVD.

In the present executive paper we provide a more synthetic companion document addressed to treat physicians and particularly to Cardiology specialists with the aim to increase knowledge on the relationships between obesity and CVD, to raise the perception of this condition which is currently insufficient and to support the clinical practice management.

## Pathophysiological mechanisms of cardiovascular diseases in obesity

Different pathophysiological mechanisms are involved in the development of CVD in obese patients (Table 1).

**Table 1:** Obesity and cardiovascular diseases: summary of pathophysiological mechanisms.

Condition	Involved mechanisms
Hypertension	Endothelial dysfunction, reduced NO, increased levels of endothelial growth factor, plasminogen-1 and thromboxane A2 increasing peripheral vascular resistance and arterial stiffness
Atherosclerosis	Visceral obesity associated to a rapid progression of coronary calcifications and to a greater vulnerability of atherosclerotic plaques
Heart failure	Neurohormonal imbalance, increased production of ROS, inflammatory mediators, leptin, resistin, visfatin and adipsin and reduced synthesis of adiponectin
Diabetes	Activation of NF-*κ*B, p38 and MAPK pathways, increased levels of IFN *γ*, TNF*α*, MCP-1, IL-1β and IL-6 and adipokines leading to insulin resistance and of concomitant metabolic alterations
Venous thromboembolism	Elevated levels of prothrombotic molecules including Factor VII, fibrinogen and tissue factor and increased expression of plasminogen activator inhibitor-1 (PAI-1)
Pulmonary hypertension	Elevated levels of cytokines, TNF-α, and interleukins, IFN γ, insulin resistance and oxidative stress

See the text for abbreviations.

**Figure 1:**
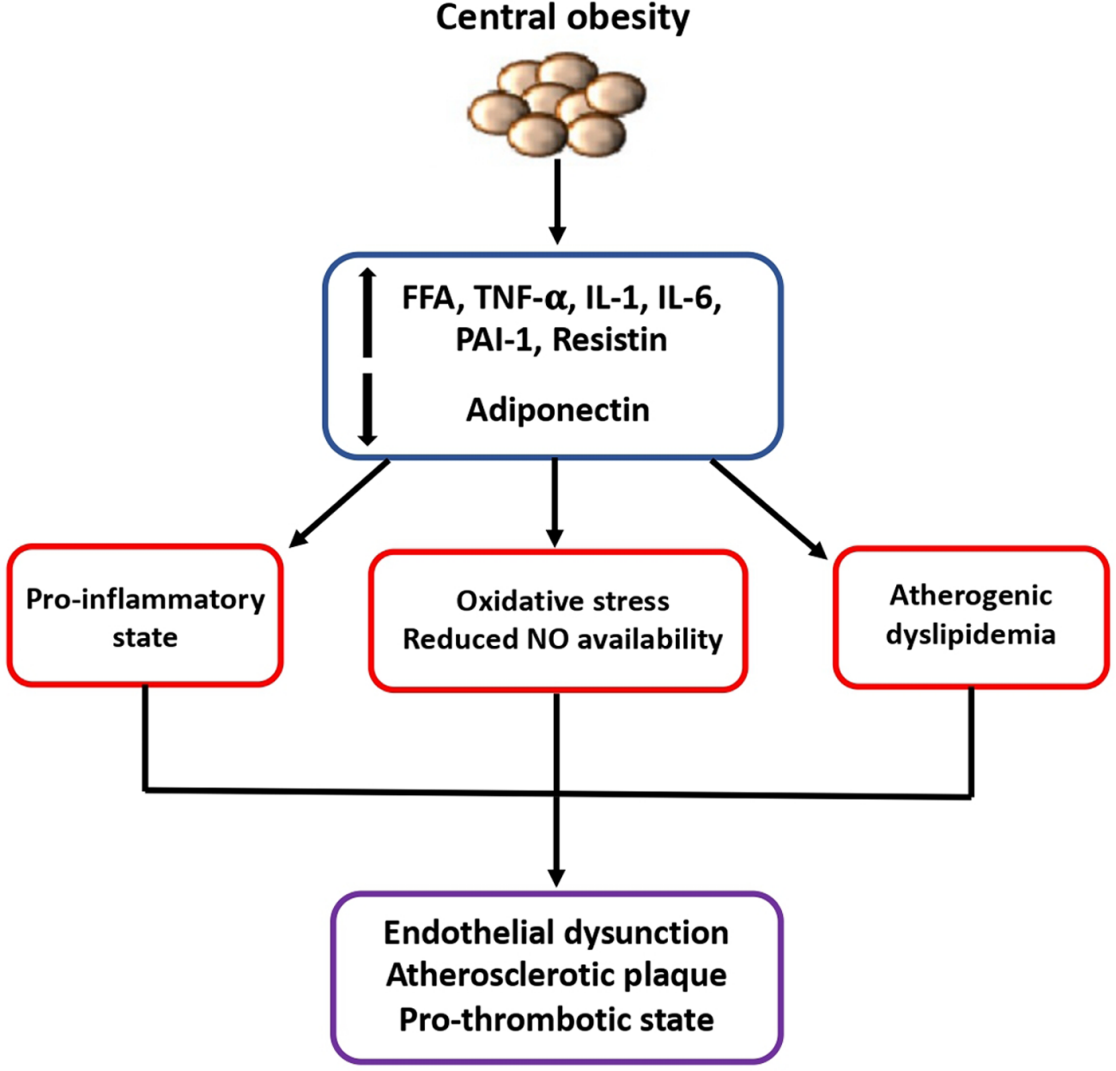
Mechanisms of higher susceptibility to cardiovascular diseases in obese patients. The expansion of visceral adipose tissue is associated to a dysregulated secretion of adipokines and other inflammatory cytokines which contribute to the development of insulin resistance, endothelial dysfunction, and prothrombotic state, finally leading to increased susceptibility to cardiovascular diseases. FFA, free fatty acid; IL-1, interleukin-1; IL-6, interleukin-6; NO, nitric oxide; PAI-1, plasminogen activator inhibitor-1; TNF, tumor necrosis factor.

First of all, it is today well recognized that the expansion of the visceral adipose tissue is associated to a dysregulation of adipokines secretion, of mitochondrial function and of lipid and glucose metabolism. Concomitant development of insulin resistance, endothelial dysfunction, sympathetic nervous system activation, increased vascular resistance and inflammatory and prothrombotic state which are promoted by obesity may definitely contribute to a higher susceptibility to CVD (5) (Figure 1).

## Obesity and diabetes

Intracellular lipid accumulation promotes the expression of toll-like-receptor-4 (TLR4) in adipocyte cells and resident macrophages, favoring the activation of NF-*κ*B, p38 and MAPK pathways, thus increasing the production of reactive oxygen species (ROS) and the secretion of inflammatory cytokines (6). Indeed, in inflammed adipose tissue T-lymphocytes express increased interferon- *γ* (IFN *γ*) levels which stimulate the production of other inflammatory cytokines including tumor necrosis factor-α (TNF*α*), monocyte chemoattractant protein-1 (MCP-1), interleukins 1*β* and 6 (IL-1β and IL-6) that recruit monocytes and mature macrophages in a vicious circle leading to hypoxia and cellular death (7, 8). Moreover, dysregulated adipose tissue produces different adipokines, including leptin, resistin, lipocalin 2, adiponectin, apelin and fibroblast growth factor-21 (FGF-21), which play an important role in the development of insulin resistance and of concomitant metabolic alterations in obese subjects (9). Insulin resistance, in turn, promotes the development and progression of the metabolic syndrome which by itself perpetuates and worsens the obesity status.

As a consequence, type 2 diabetes is a frequent associated condition accounting for 40% of obese patients. The Diabetes Prevention Study that investigated the effects of lifestyle modification on the risk of developing diabetes demonstrated that a 5% decrease of baseline body weight was associated to a 60% reduction in the risk of new-onset diabetes compared to subjects who did not achieve a weight loss (10). Consistently, in the Diabetes Prevention Program a 7% body weight reduction was associated to a 58% lower risk of developing diabetes at 4-year follow-up (11).

## Obesity and hypertension

Obesity causes microvascular damage corresponding to capillary number reduction and endothelial dysfunction, contributing to ROS secretion, free fatty acid (FFA) release, increased vascular resistances and hypertension (12).

A study conducted on 3,216 subjects has shown that 44% of hypertensive subjects were obese, whereas this percentage was only 11% among normotensive individuals (13).

Other studies demonstrated a linear relationship between body weight and blood pressure (BP) levels, corresponding to a 20%–30% increase in the risk of hypertension for each 5% rise in body weight (14). Among the proposed pathophysiological mechanisms, the increased levels of circulating FFA, angiotensin-II and leptin have been shown to play an important role (15). Obesity is indeed characterized by an increase in heart rate and in tubular sodium and water reabsorption, resulting in volume overload and increased BP levels (16). Moreover, the inflammatory status is associated with endothelial dysfunction, reduced nitric oxide production and increase in angiogenetic factors such as endothelial growth factor, plasminogen-1 and thromboxane A2, which contribute to increased peripheral vascular resistance, arterial stiffness and hypertension (17–19). Several studies have demonstrated that hypertension is a principal mediator of CV sequalae of obesity (20). Otherwise, a weight loss of 8 Kg is associated to a reduction of left ventricular wall thickness in mildly obese patients with hypertension (21).

## Obesity and atherosclerotic disease

A large body of evidence supports the association between obesity and development of major CV events, including myocardial infarction (MI), heart failure (HF) and sudden cardiac death (22–24).

In obese patients the development of atherosclerosis starts earlier and has a quicker progression than in individuals with normal body weight. Pathological studies have also shown that visceral obesity is associated to a greater vulnerability of coronary plaques (22).

In a meta-analysis of 6 studies, which included 1,593 obese subjects with documented coronary artery disease, increased mortality risk was associated to the excess of visceral obesity, defined as augmented waist circumference and waist-hip ratio, rather than to BMI (25). In the Health, Aging and Body composition study, visceral obesity was associated to an increased incidence of MI in women aged between 70 and 79 years at a 4.6-year follow-up (26). The CARDIA (Coronary Artery Risk Development in Young Adults) study has demonstrated a significant relationship between the time of exposure to excessive visceral adiposity and the presence and progression of coronary calcifications (27).

Other studies have shown that the accumulation of ectopic cardiac fat at pericardial and epicardial levels might be involved in the development of coronary atherosclerosis (28, 29). In the Multi-Ethnic Study of Atherosclerosis pericardial fat has emerged as a predictive factor for coronary events with an additive role to other traditional risk factors (30).

A 10 kg increase in body weight is associated with a 12% higher risk of coronary artery disease, worsened by the frequent coexistence of microvascular dysfunction (21). In a meta-analysis conducted on 300,000 subjects within the total of 18,000 registered acute coronary events, these were significantly more frequent among obese and overweight individuals (31).

In addition, for each kg/m2 of BMI above the normal range the risk of ischemic and hemorrhagic stroke increases by 4% and 6% respectively (21).

## Obesity and heart failure

**Figure 2:**
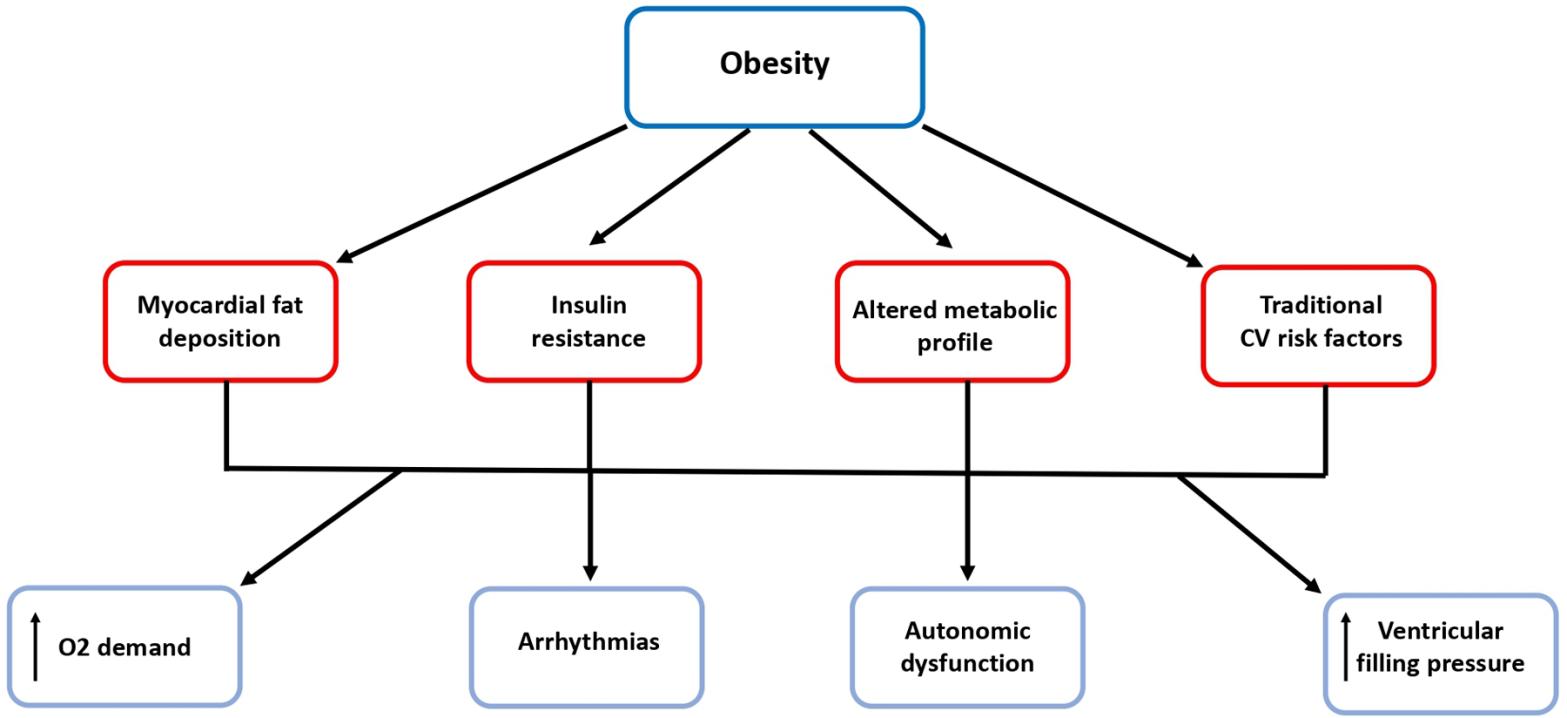
Obesity predisposes to arrhythmias and heart failure. Beside the relationship with traditional risk factors, obesity is associated with myocardial fat deposition, insulin resistance and altered metabolic profile, which contribute to the development of atrial and ventricular remodeling, systolic and diastolic dysfunction and to increased ventricular filling pressures and finally to arrhythmias and overt HF. CV, cardiovascular; O2, oxygen.

Obesity contributes to the development of atrial and ventricular remodeling, systolic and diastolic dysfunction and to an increase in ventricular filling pressures and pulmonary pressures which result in subclinical organ damage and then may progress to overt HF (21–23). Tachycardia, excess blood volume and increased vascular resistance and cardiac overload are common CV “finger-prints” in obese subjects. Moreover, increased fibrosis, reduced conduction velocity, epicardial fat and fatty infiltration represent detrimental factors to atrial and ventricular electrophysiologcal properties which may promote a proarrhythmic substrate (32). These morphological and functional changes often lead to atrial fibrillation which *per se* represents another patho-physiological mechanism of HF as well as a condition predisposing to stroke (23) (Figure 2).

A key role in the pathogenesis of HF is carried out by the neurohormonal imbalance consisting in the hyperactivation of the sympathetic nervous and renin angiotensin aldosterone systems, by the increased production of ROS, inflammatory mediators including IL-6, TNF-α, C-reactive protein, leptin, resistin, visfatin and adipsin and by the reduced synthesis of adiponectin (33).

The excess of visceral adiposity causes an increased degradation of natriuretic peptides (NPs) (34), which are produced by the heart in conditions of volume and pressure overload and exert different protective functions in HF with natriuretic, diuretic, anti-fibrotic and anti-remodeling actions counterbalancing the detrimental neurohormonal hyperactivation. NPs also promote lipolysis and synthesis of adiponectin (35).

In obese subjects HF develops about 10 years earlier than in those with normal body weight. The risk of HF is increased respectively by 70% after 20% and 90% after 30 years lived in a condition of obesity (36).

A sub-analysis of the CHARM study showed that the 75% of patients affected by HF before an age of 40 years were obese or overweight (37).

Among patients affected by HF the proportions of obese and overweight subjects range between 32% and 49% and 31%–40%, respectively, and a 10% of HF cases is directly referable to obesity (21).

In addition, every 2 years lived in a condition of obesity the risk of CV mortality is increased by 7% (38). Consistently, data from the Framingham Heart Study have shown a linear relationship between the duration of obesity and mortality irrespective of BMI and concomitant risk factors. The incidence of sudden cardiac death is 40-fold higher in obese subjects as a consequence of increased electrical irritability, ion channel remodeling, reduction of connexin proteins (39) and impaired sympathetic-vagal balance and of more frequent and complex ventricular arrhythmias even in the absence of an overt HF condition (35).

## Obesity and obstructive sleep apnea syndrome

Obesity represents one of the most important reversible risk factors for obstructive sleep apnea syndrome (OSAS), accounting for 41% and 58% of total and moderate-to-severe cases, respectively (40).

OSAS is characterized by repeated episodes of upper airway obstruction during sleep, resulting in repetitive hypoxemia and intermittent pauses in breathing causing oxygen desaturation, arousal from sleep and excessive daytime sleepiness (41). The prevalence of OSAS is three-fold higher among obese subjects because of airways narrowing due to fat accumulation, increased mechanical loading of the respiratory system and reduced functional residual capacity (42). As a consequence, OSAS may contribute to increase the obesity-related risk of hypertension, stroke, CVD and sudden death (43).

## Obesity, venous thromboembolism and pulmonary hypertension

Obesity has been associated with an increased risk of venous thromboembolism (VTE), consisting in pulmonary embolism and deep venous thrombosis (44). Different factors contribute to the development of VTE in obese patients, including elevated levels of prothrombotic molecules such as Factor VII, fibrinogen and tissue factor, an increased expression in visceral fat of plasminogen activator inhibitor-1 (PAI-1) resulting in impaired fibrinolysis, reduced venous return and physical activity (45, 46). Moreover, obese patients are more prone to develop pulmonary hypertension after a first PE (47). In such a context, systemic and local inflammation with elevated levels of cytokines, TNF-α, and interleukins, IFN *γ*, insulin resistance and oxidative stress play a role in exacerbating the vascular remodeling process involved in pulmonary hypertension (47).

## Obesity and outcomes of COVID-19: the lessons learned

In the last 3 years the coronavirus disease 2019 (COVID-19), caused by the severe acute respiratory syndrome coronavirus 2 (SARS-CoV-2), has dramatically changed the priorities and the use of available resources by the national healthcare systems (48, 49).

A bidirectional link exists between obesity and COVID-19. Due to the lockdown measures introduced in the early phases of the pandemic aimed at reducing SARS-CoV-2 transmission a significant increase in the incidence of obesity has been documented, this phenomenon being described as “covibesity” (50). On the other hand, a large body of evidence has shown that obesity is a determinant factor for the severity of COVID-19.

In a retrospective study conducted on 124 patients who were admitted to intensive care unit (ICU) for severe COVID-19 the 75.8% were obese (51). In another study on 3,615 patients, obesity has emerged as a fundamental prognostic factor for a severe course of COVID-19. Patients aged <60 years with a BMI >30 kg/m² had a 2-fold higher risk of being hospitalized and to develop a severe disease compared to those with normal body weight. In a prospective cohort study that enrolled 7 million individuals has demonstrated that a BMI >23 kg/m² is associated to a worse prognosis in patients affected by severe COVID-19, particularly in those aged <40 years and Black people. A linear relationship has been detected between BMI increase and the risk of hospitalizations, mortality and ICU admission. The risk of being admitted to ICU was 4-fold higher in patients with severe obesity (BMI >35 kg/m²) (52).

The hypothesized pathophysiological mechanisms include the secretion of adipokines, chemokines and cytokines, the impaired qualitative and quantitative response of immune cells in the adipose tissue, with a significant reduction of Th2 and Treg cells, of M2 macrophages and an increase of pro-inflammatory T CD8+ and M1 macrophages (53).

For these reasons and particularly in view of the high toll paid by obese patients during the course of SARS-COV-2 infection, as well as during the course of other viral or bacterial infections, it appears reasonable to consider the obese as a frail population which needs specific measures of prevention and care.

## Pharmacological and non-pharmacological strategies

Lifestyle changes represent the first step to achieve and maintain an effective body weight reduction.

Energy restriction is the cornerstone of weight loss, particularly when associated to physical activity. With this aim, several types of dietetic strategies may be suggested consisting in hypocaloric diets, Mediterranean diet, high-protein diets to preserve lean muscle mass and enhance satiety, low or very low carbohydrate diets, moderate carbohydrate diets and low-fat diets, intermittent fasting or time-restricted eating diets. Among the proposed strategies, the benefits of the Mediterranean diet tend to persist over time without an increased risk of ketogenesis (54, 55).

However, this type of intervention is often not sufficient and additional pharmacological and not-pharmacological measures are required (54).

For many years bariatric/metabolic surgery (BMS), defined as the procedures inducing loss of body weight throughout the modification of gastrointestinal physiology, has represented the only available and more effective strategy. BMI ≥40 kg/m^2^ or BMI ≥35 kg/m^2^ with comorbidities are considered the threshold for surgery by international guidelines. The dominant procedures are sleeve gastrectomy and roux-en-Y gastric bypass, accounting for approximately 90% of all operations performed worldwide with efficacious mid- and long-term outcomes. Other approaches include biliopancreatic diversion with duodenal switch, one-anastomosis gastric bypass and the less invasive adjustable gastric banding (56). BMS procedures produce a 14%–25% body weight reduction associated to a significant lower risk of hypertension, diabetes, non-alcoholic fatty liver disease and mortality. However, BMS remains an underused tool, prescribed only to the 1%–2% of subjects who may benefit, probably due to inadequate information and support from healthcare systems and to the risks associated with surgery (57, 58).

In the last few years different pharmacological strategies have been introduced in clinical practice.

Liraglutide, a glucagon-like peptide-1 receptor agonist (GLP1-RA) already approved for the treatment of type 2 diabetes has been recently introduced at the dosage of 3 mg for obese subjects with associated comorbidities such as hypertension, dyslipidemia and obstructive sleep apnea syndrome (59–63). Beside the glucose-lowering effect related to the stimulation of insulin secretion, liraglutide slows gastric emptying and increases hypothalamic sense of satiety, stimulating pro-opiomelanocortin (POMC) neurons (64). In the SCALE Obesity and Prediabetes the loss of body weight produced by the treatment with liraglutide has been maintained for 3 years and has been associated to a 80% reduction of the risk of developing diabetes and to an improvement of metabolic profile (65). Moreover, a body weight loss of 7.8 Kg was associated with a 12.5 mmHg reduction of systolic BP (65).

The association of naltrexone, used for the treatment of depression and nicotine addiction, with bupropion, used for addiction to opioids and alcohol, has been demonstrated to reduce body weight by acting on hypothalamic nucleus arcuatus and on the dopaminergic mesolimbic system (66–70). In particular, bupropion stimulates POMC neurons to release *α*-melanocyte-stimulating hormone, whereas naltrexone blocks the negative feedback produced by the action of *β*-endorphins on POMC neurons. As a consequence, naltrexone enhances the action of bupropion increasing energetic expenditure and reducing appetite (71).

Semaglutide, another long lasting GLP1-RA, has been recently approved at the dosage of 2.4 mg. The STEP (Semaglutide Treatment Effect in People with obesity) study and its sub-analyses have generated great enthusiasm due to the greater loss of body weight compared to previously experimented drugs (72–76). Treatment with semaglutide produced a 5% reduction of body weight in >90% of subjects and a 20% reduction in about 35%, these results being comparable to those achieved with BMS. Consistently, patients who received semaglutide improved their BP control and their exercise performance (72–76). The Semaglutide Effects on Heart Disease and Stroke in Patients with Overweight or Obesity (SELECT) study is testing the superiority of semaglutide 2.4 mg subcutaneously once weekly compared to placebo in preventing major adverse cardiovascular events in patients with established CVD and overweight or obesity but without diabetes. As such, SELECT has the potential for advancing new approaches to CVD risk reduction while targeting obesity (77).

Growth differentiation factor 15 (GDF15), a distant member of the transforming growth factor-*β*, has been demonstrated to bind glial cell-derived neurotrophic factor family receptor alpha-like (GFRAL) reducing the intake of high-fat diets in animal models, to recruit the receptor tyrosine kinase (RET) contributing to weight loss and to improve glycemic control (78). On the basis of these evidence, long-acting analogues of GDF15 are currently under investigation and might represent a future interesting therapeutic option for obese patients.

## Conclusions

In this document the SIPREC Committee aimed to provide an extended update of the role of obesity not only as an amplifier of traditional risk factors, but also as an independent complex chronic and recurrent condition whose pathophysiological aspects needs further studies and whose management deserves specific and targeted pharmacological and non-pharmacological strategies.

The clinical appraisal of obesity must increase, as it is deserved by a chronic disease which carries a heavy burden of CV and metabolic consequences. More structured lifestyle advice and new available medications should be systematically prescribed whenever appropriate and as early as possible in the clinical practice to reduce the consequent disease burden and the metabolic and CV sequelae of this condition.

Our auspices are to promote an increasing interest in the medical community and the adoption of early and effective tailored treatment strategies to fight this emerging disease.

## Data Availability

The original contributions presented in the study are included in the article/Supplementary Material, further inquiries can be directed to the corresponding author/s.

## Appendix

Alessio Basolo and Guido Salvetti, Obesity and Lipodystrophy Center, Endocrinology Unit, University Hospital of Pisa, Pisa, Italy; Simonetta Bellone and Roberta Ricotti, Department of Health Sciences, University of Piemonte Orientale, Novara, Italy; Vanessa Bianconi, Massimo R. Mannarino and Matteo Pirro, Unit of Internal Medicine, Angiology and Arteriosclerosis Diseases, Department of Medicine, University of Perugia, Perugia, Italy; Agostino Consoli, Department of Medicine and Aging Sciences and Centro Scienze dell'Invecchiamento-Medicina Traslazionale (CeSI-MeT), University G. D'Annunzio, Chieti, Italy; Maurizio De Luca, Division of General Surgery, Castelfranco and Montebelluna Hospitals, Treviso, Italy; Rita Del Pinto, Claudio Ferri and Giovambattista Desideri, Department of Life, Health & Environmental Sciences, University of L'Aquila, L'Aquila, Italy; Leonarda Galiuto, Department of Clinical and Molecular Medicine, Sapienza University of Rome, Rome, Italy; Guido Grassi and Gino Seravalle, University of Milano-Bicocca, Milan, Italy; Elisa Lodi and Maria Grazia Modena, University of Modena and Reggio Emilia, Modena, Italy; Maria Chiara Meucci, Department of Cardiovascular and Pulmonary Sciences, Catholic University of the Sacred Heart Rome, Italy; Carmine Morisco and Bruno Trimarco, Department of Advanced Biomedical Sciences, Federico II University of Naples, Naples, Italy; Giulio Nati, General Practitioner; Speranza Rubattu, Department of Clinical and Molecular Medicine, Sapienza University of Rome, Rome, Italy and IRCCS Neuromed, Pozzilli (IS), Italy; Saula Vigili de Kreutzenberg, Department of Medicine-DIMED, University of Padova, Padua, Italy; Roberto Volpe, Health and Safety Unit (SPP), National Research Council (CNR), Rome, Italy.

Italian Society of Cardiology [SIC], Italian Society of Diabetology (SID), Italian Society of Internal Medicine [SIMI], Italian Society of Arterial Hypertension [SIIA], Italian Society for the Study of Atherosclerosis [SISA], Italian Society of Nephrology [SIN], Italian Society of Obesity [SIO], Italian Society of Digital Health and Telemedicine [SIT], Italian Society of Nutraceutics [SINut], Italian Association of Clinical, Preventive and Rehabilitation Cardiology (AICPR), Italian Society of Gerontology and Geriatrics [SIGG], the Mediterrean Diet Foundation [FDM].
